# Efficacy and safety profile of deep responders to carfilzomib-based therapy: a subgroup analysis from ASPIRE and ENDEAVOR

**DOI:** 10.1038/s41375-020-01049-5

**Published:** 2020-10-16

**Authors:** Katja Weisel, Maria-Victoria Mateos, Francesca Gay, Michel Delforge, Gordon Cook, Zsolt Szabo, Renaud Desgraz, Lucy DeCosta, Philippe Moreau

**Affiliations:** 1 Department of Oncology and Hematology, University Medical Center of Hamburg-Eppendorf, Hamburg, Germany; 2 Institute of Biomedical Research of Salamanca (IBSAL), Cancer Research Center-IBMCC (USAL-CSIC), and Hematology Department, University Hospital of Salamanca, Salamanca, Spain; 3 Myeloma Unit, Division of Hematology, Azienda Ospedaliero Universitaria Città della Salute e della Scienza, University of Turin, Turin, Italy; 4 Department of Hematology, University Hospital (UZ) Leuven, Leuven, Belgium; 5 Department of Haematology, Leeds Cancer Centre, St James’s University Hospital, Leeds, UK; 6 Amgen (Europe) GmbH, Rotkreuz, Switzerland; 7 Global Biostatistical Science, Amgen Ltd, Cambridge, UK; 8 Hematology Department, University Hospital Hôtel-Dieu, Nantes, France

**Keywords:** Myeloma, Haematological cancer

## Abstract

To understand the profile of best responders (complete response or better [≥CR]) to carfilzomib, we described the characteristics, progression-free survival (PFS), overall survival (OS) data, and the safety of patients who achieved ≥CR to carfilzomib-based treatment in ASPIRE and ENDEAVOR. In post hoc analyses from ASPIRE and ENDEAVOR, median PFS and OS were longer for ≥CR patients versus those who achieved a very good partial response or partial response (VGPR/PR). In the carfilzomib arm of ASPIRE, median PFS was 50.4 months for ≥CR versus 22.1 months for VGPR/PR; median OS was 67.0 versus 44.2 months, respectively. In the carfilzomib arm of ENDEAVOR, median PFS was 34.0 for ≥CR versus 20.4 months for VGPR/PR; median OS was non-estimable. Despite the longer treatment duration, fewer patients with ≥CR versus VGPR/PR experienced treatment-emergent adverse events that led to discontinuation of carfilzomib-based treatment in ASPIRE or ENDEAVOR. Low serum lactate dehydrogenase was the only factor associated with achieving ≥CR vs patients not achieving CR in ASPIRE in multivariate regression analyses. No association was found between cytogenetic risk status and reaching ≥CR. Carfilzomib treatment may lead to rapid and deep responses, irrespective of most patient characteristics.

## Introduction

Multiple myeloma (MM) is a currently incurable hematological malignancy characterized by proliferation of malignant plasma cells in the bone marrow [1–3]. The disease has a relapsing course, which means that patients typically receive multiple lines of treatment [4, 5]. Recently, there has been an expansion in the number of pharmacological treatments available for patients with either newly diagnosed or relapsed/refractory MM (RRMM), which has improved patient outcomes [4, 6, 7]. Carfilzomib is a proteasome inhibitor that is approved in several countries; in Europe and the United States of America, it is indicated for use in patients with RRMM in combination with either lenalidomide and dexamethasone or dexamethasone alone [8–10]. Carfilzomib was approved based on results of the pivotal phase 3 randomized controlled trials ASPIRE (NCT01080391) and ENDEAVOR (NCT01568866) in patients with RRMM, which demonstrated superior progression-free survival (PFS [primary outcome]) and significant overall survival (OS [secondary outcome]) improvement for the carfilzomib-based treatments (carfilzomib, lenalidomide and dexamethasone [KRd; ASPIRE], or carfilzomib and dexamethasone [Kd; ENDEAVOR]) compared with the comparator regimens (lenalidomide and dexamethasone [Rd; ASPIRE], or bortezomib and dexamethasone [Vd; ENDEAVOR]) [8, 9, 11–14]. At the prespecified interim analysis, median PFS was 26.3 versus 17.6 months in the KRd versus Rd arm, respectively [11], and 18.7 versus 9.4 months in the Kd versus Vd arm, respectively [12]. In the intent-to-treat analyses, median OS was 48.3 months in the KRd arm versus 40.4 months in the Rd arm (hazard ratio [HR]: 0.79), and 47.8 versus 38.8 months in the Kd versus Vd arm (HR: 0.76) [13, 14]. Furthermore, significantly more patients treated with carfilzomib-based regimens achieved a complete response or better (≥CR) than those treated with the comparator regimens (KRd vs Rd, 31.8 vs 9.3%, *p* < 0.001 [11]; Kd vs Vd, 13 vs 6%, *p* = 0.001) [12]. The efficacy benefits of carfilzomib-based versus comparator arm treatments have been maintained across subsequent analyses of both studies [14–20].

Given the number of agents now approved for the treatment of RRMM, treatment decisions are becoming increasingly complex [7, 21–24]. While the choice of treatment regimen at relapse typically takes account of patient, disease and treatment characteristics (e.g., performance status, comorbidities, tumor burden, cytogenetics, previous treatment responses and toxicities), there is no validated strategy for the identification of patients who are likely to respond well to a given treatment at relapse [23, 25–28]. Factors such as cytogenetics, serum albumin, serum lactate dehydrogenase (LDH) and β2-microglobulin are established prognostic variables in patients with newly diagnosed MM; however, the role of these factors in predicting prognosis in RRMM has been less well studied [25–32]. Recently, a risk stratification algorithm has been developed to help physicians predict survival outcomes in patients starting second-line treatment, which incorporates 16 predictors relating to patient frailty and disease aggressiveness at diagnosis and following first-line treatment [33].

The aim of the current post hoc, exploratory analyses was to describe the characteristics of patients who achieved a best response (namely ≥CR) to carfilzomib-based treatment to help support treatment decisions in clinical practice. For this purpose, we analyzed patient-level PFS and OS data for patients who achieved ≥CR to carfilzomib-based treatment in ASPIRE and ENDEAVOR, and sought to identify potential predictors of ≥CR.

## Methods

These post hoc analyses included data from patients with extended follow-up in ASPIRE (data cut-off date: 28 April 2017) [14] and ENDEAVOR (data cut-off date: 19 July 2017 [13]). Data from each study were described separately without any direct comparison made between studies.

### ASPIRE and ENDEAVOR study designs

Full details of ASPIRE and ENDEAVOR have been described elsewhere [11, 12]. Briefly, ASPIRE was an open-label, head-to-head phase 3 study in adults with relapsed MM who had received one to three previous treatments. Patients were randomized (1:1) to receive KRd or Rd as previously described [11]. ENDEAVOR was an open-label, head-to-head phase 3 study in adults with RRMM who had received one to three previous treatments and had achieved at least a partial response to one previous treatment. Patients were randomized (1:1) to receive Kd or Vd as previously described [12]. In both studies, patients were treated until withdrawal of consent, disease progression or unacceptable toxicity. An independent review committee assessed disease progression and response to treatment in both studies as previously described [11, 12]; this assessment of response was used to categorize the patient cohorts for the present analysis. PFS was derived using the investigators’ disesase assessments which allow for an extended follow up time.

### Patient subgroups included in the present analyses

The subgroup of patients achieving CR or better (≥CR subgroup) was selected to represent the best responders in each treatment arm. The ≥CR subgroup provided an adequate sample size for this analysis. A second subgroup was identified consisting of patients who achieved very good partial response (VGPR) or partial response (PR) (VGPR/PR subgroup). The ≥CR and VGPR/PR subgroups together represented the total group of patients with an overall response in each treatment arm of ASPIRE and ENDEAVOR. In the multivariate analyses, carfilzomib-treated patients were considered as achieving ≥CR versus not (i.e., a best response of VGPR, PR, minimal response [MR], stable disease [SD], or progressive disease [PD]).

### Analyses

#### Survival analysis

Kaplan–Meier (KM) estimates of PFS and OS by response status (≥CR vs VGPR/PR) were obtained using a naïve analysis approach.

#### Exposure to study treatment and safety analyses

Exposure to carfilzomib-based treatment (number of cycles started or number of cycles patient dosed), the exposure-unadjusted incidence of treatment-emergent adverse events (TEAEs), and the exposure-adjusted risks of grade 3 or higher TEAEs of interest were described for the ≥CR and VGPR/PR subgroups of the safety population of the KRd and Kd arms of ASPIRE and ENDEAVOR, respectively. Exposure-adjusted risks were calculated by dividing the total number of patients with events by total person time; total person time was the sum of time to first TEAE for all patients in each subgroup. For patients who did not experience a TEAE, the entire time exposed to study treatment was considered in the sum. TEAEs were defined as any adverse event with an onset date between the first dose and 30 days after the last dose of any study drug.

#### Multivariate analysis for a best response (≥CR)

For carfilzomib-treated patients (KRd or Kd), multivariate logistic regression models were used to assess the association between patient-, disease-, and prior treatment-related factors and the likelihood of achieving ≥CR (vs patients who did not achieve a CR, including VGPR, PR, MR, SD, and PD). Eleven factors were included in the regression models: number of previous treatment lines (≥2 vs 1); Eastern Cooperative Oncology Group (ECOG) performance status (1–2 vs 0); serum LDH (>360 U/l vs ≤360 U/l); cytogenetics (high vs standard risk/not reported [NR]); serum calcium (>2.75 vs ≤2.75 mmol/l); refractory status to immediately previous regimen (yes vs no); age (≥65 vs <65 years); bone marrow plasma cell count (≥70 vs <70%); serum β2-microglobulin level (≥3.5 vs <3.5 mg/l); serum thrombocyte count (≤100 × 10^9^ cells/l vs >100 × 10^9^ cells/l); and serum albumin level (<3.5 vs ≥3.5 g/dl). Serum LDH was not included in the model for ENDEAVOR because no patients treated with Kd had a serum LDH greater than the threshold value used in this analysis (serum LDH > 360 U/l). High-risk cytogenetics groups have been defined previously [11, 12]. These factors and thresholds have been shown to be clinically relevant and are generally available in trial datasets [33].

## Results

Patient numbers in the ASPIRE intention-to-treat (ITT) population were: KRd, *n* = 396; Rd, *n* = 396 [11]. In ENDEAVOR they were: Kd, *n* = 464; Vd, *n* = 465 [12]. For these post hoc analyses we used the respective data cut-off in each study leading to a median follow-up of 48.8 months for PFS and 67.1 months for OS for carfilzomib-treated patients from ASPIRE [14], and 44.3 months for OS for carfilzomib-treated patients from ENDEAVOR [13]. In the carfilzomib arms, patients from ASPIRE were treated for a median of 72 weeks (i.e., 18 cycles; per protocol, carfilzomib was stopped after 18 cycles) [14] and patients from ENDEAVOR for 48 weeks (interquartile range [IQR]: 24.1–88.7 weeks), which equated to a median of 12 cycles (IQR: 6–22 cycles). In total, 126 patients (31.8%) and 58 patients (13%) treated with KRd and Kd, respectively, had achieved ≥CR. The median time to ≥CR for patients treated with KRd in ASPIRE was 6.7 months (min, max: 1.4, 30.2 months) [15] and for those treated with Kd in ENDEAVOR was 6.8 months (min, max: 2.0, 20.8 months) (previously unpublished), with 75% of patients achieving the ≥CR within a year of treatment on both studies (KRd: 11.8 months; Kd: 10.9 months).

Baseline characteristics for patients in the ≥CR, compared with VGPR/PR subgroups and overall patients from the carfilzomib arms of ASPIRE and ENDEAVOR are provided in Table 1. For both studies, patients who achieved ≥CR differed from VGPR/PR patients in that more patients in the ≥CR subgroup had an ECOG performance status of zero (KRd: 46.8 vs 41.6%, respectively; Kd: 65.5 vs 45.3%, respectively), and fewer patients had received three or more previous lines of treatment (KRd: 19.0 vs 24.2%, respectively; Kd: 12.1 vs 16.8%); Specific characteristics of the patients treated with KRd in ASPIRE who achieved ≥CR was that more patients had an ISS stage 3 (54.0 vs 41.6% in VGPR/PR) and they presented with a lower median serum LDH (223.0 U/l [minimum–maximum: 84.0–743.0 U/l] vs 230.5 [82.0–1205.0] in VGPR/PR). Of the patients treated with Kd in ENDEAVOR who achieved a ≥CR, they were characterized by a younger median age (62 years [35–81] vs 66 [36–89] in VGPR/PR), a lower median serum β2-microglobulin level (3.24 mg/l [1.65–11.70] vs 3.51 [1.44–24.20] in VGPR/PR); more patients were at high-risk cytogenetics (25.9 vs 18.5% in VGPR/PR); more patients had an ISS stage 1 (55.2 vs 48.3% in VGPR/PR); more patients had undergone HSCT previously (65.5 vs 52.7% in VGPR/PR); and fewer patients were refractory to lenalidomide (15.5 vs 20.1% in VGPR/PR). Of note, 11.1% (14/126) and 25.9% (15/58) of patients who received carfilzomib-based treatment and achieved ≥CR in ASPIRE and ENDEAVOR, respectively, had high-risk cytogenetics.

**Table 1:** Baseline characteristics^a^ according to response (best response [≥CR] vs VGPR/PR) vs total in the carfilzomib arms of ASPIRE (KRd) and ENDEAVOR (Kd).

Baseline characteristics^a^	ASPIRE (KRd)			ENDEAVOR (Kd)		
	≥CR (*n* = 126)	VGPR/PR (*n* = 219)	Total KRd (*N* = 396)	≥CR (*n* = 58)	VGPR/PR (*n* = 298)	Total Kd (*N* = 464)
Median age, years (range)	65 (38–85)	63 (40–87)	64 (38–87)	62 (35–81)	66 (36–89)	65 (35–89)
Males, *n* (%)	68 (54.0)	119 (54.3)	215 (54.3)	29 (50.0)	142 (47.7)	240 (51.7)
ECOG performance status, *n* (%)						
0	59 (46.8)	91 (41.6)	165 (41.7)	38 (65.5)	135 (45.3)	221 (47.6)
1	56 (44.4)	107 (48.9)	191 (48.2)	19 (32.8)	140 (47.0)	211 (45.5)
2	11 (8.7)	21 (9.6)	40 (10.1)	1 (1.7)	23 (7.7)	32 (6.9)
Mean serum β2-microglobulin level, mean, mg/l (SD)	3.89 (2.07)	4.02 (1.99)	4.06 (2.08)	4.08 (2.30)	4.34 (2.82)	4.57 (3.00)
Median serum β2-microglobulin level, median, mg/l (range)	3.40 (1.30–13.00)	3.50 (1.50–12.50)	3.50 (1.30–13.00)	3.24 (1.65–11.70)	3.51 (1.44–24.20)	3.60 (1.44–24.20)
Mean serum LDH level (U/l), mean (SD)	256.3 (117.05)	291.7 (182.25)	289.3 (176.71)	174.3 (45.6)	204.0 (137.2)	217.7 (172.0)
Median serum LDH level (U/l), median (range)	223.0 (84.0–743.0)	230.5 (82.0–1205.0)	238.0 (82.0–1241.0)	170.5 (24.0–330.0)	172.0 (75.0, 1327.0)	180.5 (24.0, 2130.0)
Cytogenetics,^b^ *n* (%)						
High-risk	14 (11.1)	24 (11.0)	48 (12.1)	15 (25.9)	55 (18.5)	97 (20.9)
t(4;14)	11 (8.7)	15 (6.8)	33 (8.3)	8 (13.8)	31 (10.4)	50 (10.8)
t(14;16)	1 (0.8)	1 (0.5)	2 (0.5)	2 (3.4)	6 (2.0)	10 (2.2)
del(17p)	3 (2.4)	13 (5.9)	22 (5.6)	5 (8.6)	20 (6.7)	40 (8.6)
Standard risk	56 (44.4)	78 (35.6)	147 (37.1)	37 (63.8)	187 (62.8)	284 (61.2)
Unknown	56 (44.4)	117 (53.4)	201 (50.8)	5 (8.6)	37 (12.4)	55 (11.9)
Missing	–	–	–	1 (1.7)	19 (6.4)	28 (6.0)
ISS stage at baseline,^c^ *n* (%)						
1	–	–	–	32 (55.2)	144 (48.3)	212 (45.7)
2	–	–	–	15 (25.9)	89 (29.9)	138 (29.7)
3	–	–	–	11 (19.0)	65 (21.8)	114 (24.6)
Study-site-reported ISS stage at initial diagnosis, *n* (%)						
1	17 (13.5)	44 (20.1)	64 (16.2)	–	–	–
2	27 (21.4)	57 (26.0)	99 (25.0)	–	–	–
3	68 (54.0)	91 (41.6)	185 (46.7)	–	–	–
Unknown	14 (11.1)	27 (12.3)	48 (12.1)	–	–	–
Previous HSCT, *n* (%)	72 (57.1)	124 (56.6)	217 (54.8)	38 (65.5)	157 (52.7)	266 (57.3)
Number of previous regimens, *n* (%)						
1	62 (49.2)	98 (44.7)	184 (46.5)	27 (46.6)	163 (54.7)	232 (50.0)
2	40 (31.7)	68 (31.1)	120 (30.3)	24 (41.4)	85 (28.5)	157 (33.8)
3	24 (19.0)	52 (23.7)	91 (23.0)	7 (12.1)	50 (16.8)	75 (16.2)
4	0	1 (0.5)	1 (0.3)	0	0	0
Previous treatment status, *n* (%)						
Received bortezomib	78 (61.9)	147 (67.1)	261 (65.9)	24 (41.4)	153 (51.3)	250 (53.9)
Refractory to bortezomib	17 (13.5)	31 (14.2)	60 (15.2)	2 (3.4)	8 (2.7)	15 (3.2)
Received lenalidomide	19 (15.1)	45 (20.5)	79 (19.9)	19 (32.8)	104 (34.9)	177 (38.1)
Refractory to lenalidomide	4 (3.2)	16 (7.3)	29 (7.3)	9 (15.5)	60 (20.1)	113 (24.4)
Received thalidomide	60 (47.6)	96 (43.8)	176 (44.4)	29 (50.0)	133 (44.6)	211 (45.5)

*≥CR* complete response or better, *ECOG* Eastern Cooperative Oncology Group, *FISH* fluorescence in situ hybridization, *HSCT* hematopoietic stem cell transplantation, *LDH* lactate dehydrogenase, *IMiD* immunomodulatory drug, *IQR* interquartile range, *ISS* International Staging System, *Kd* carfilzomib and dexamethasone, *KRd* carfilzomib, lenalidomide and dexamethasone, *MR* minimal response, *NA* not applicable, *PR* partial response, *SD* standard deviation, *VGPR* very good partial response.

^a^Data for baseline characteristics are provided if they were recorded in both ASPIRE and ENDEAVOR.

^b^Determined by FISH. In ASPIRE, the high-risk cytogenetics group comprised patients with the genetic subtypes *t*(4;14), *t*(14;16), or with deletion 17p in at least 60% of plasma cells. The standard-risk cytogenetics group comprised patients without *t*(4;14), *t*(14;16), or with deletion 17p in fewer than 60% of plasma cells [11]. In ENDEAVOR, the high-risk cytogenetics group comprised patients with genetic subtypes *t*(4;14), (14;16) in at least 10% of plasma cells, or with deletion 17p in at least 20% of plasma cells. The standard-risk cytogenetics group comprised patients without these genetic subtypes [12].

^c^Baseline was defined as the last available measurement taken before cycle 1, day 1.

### Survival analysis

**Fig. 1:**
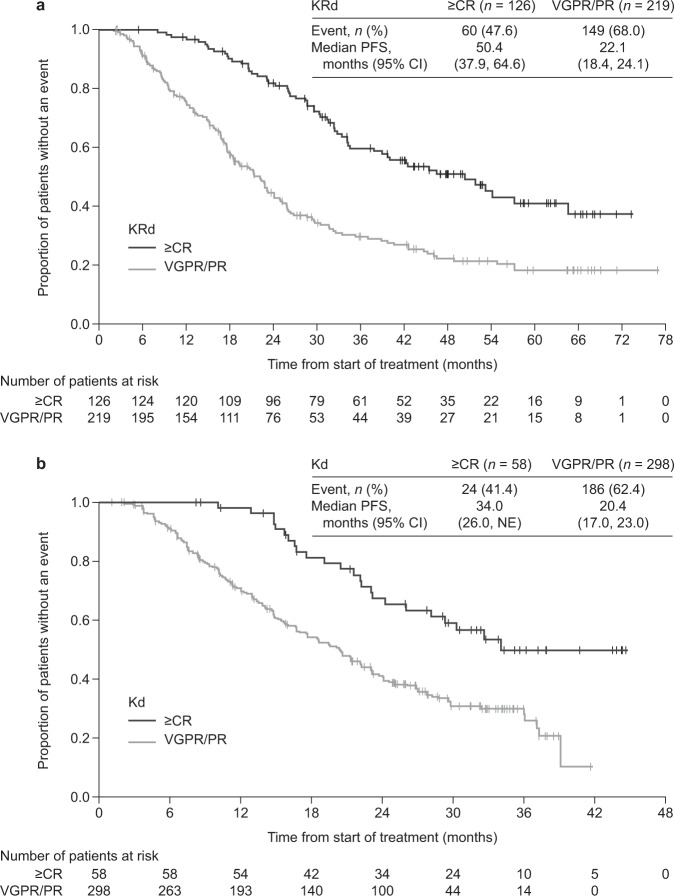
KM curves for PFS for patients who achieved a best response (≥CR) versus those who achieved VGPR/PR from **a** the KRd arm of ASPIRE and **b** the Kd arm of ENDEAVOR. KM estimates of PFS in the carfilzomib arms by response status were obtained using a naïve analysis approach. Caution is warranted when interpreting these naïve analyses owing to the absence of adjustment for bias. *≥CR* complete response or better, *CI*, confidence interval, *Kd* carfilzomib and dexamethasone, *KM* Kaplan–Meier, *KRd* carfilzomib, lenalidomide and dexamethasone, *PFS* progression-free survival, *PR* partial response, *VGPR* very good partial response.

**Fig. 2:**
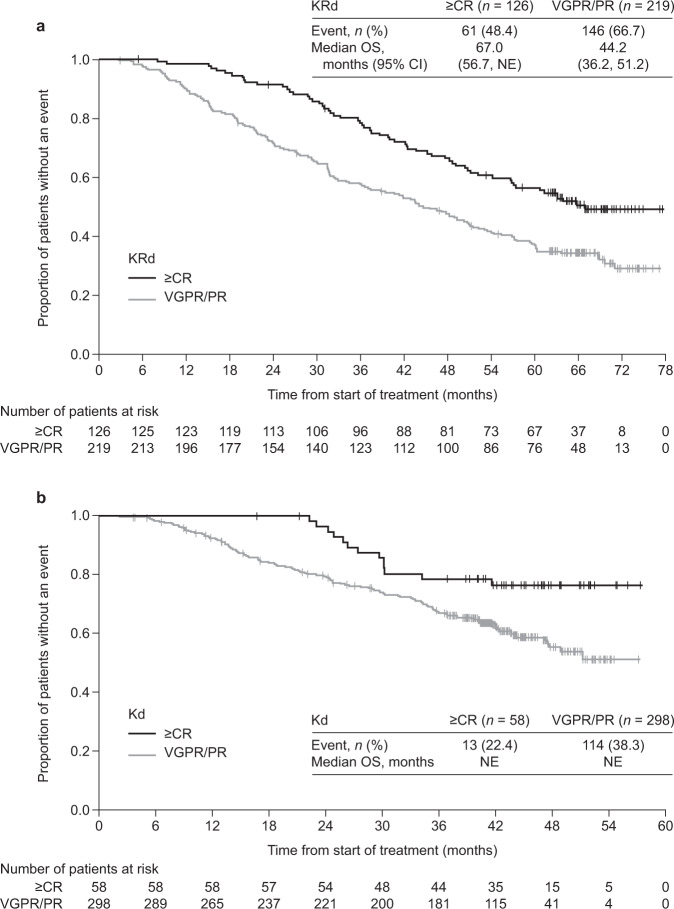
KM curves for OS for patients who achieved a best response (≥CR) versus those who achieved VGPR/PR from **a** the KRd arm of ASPIRE and **b** the Kd arm of ENDEAVOR. KM estimates of OS in the carfilzomib arms by response status were obtained using a naïve analysis approach. Caution is warranted when interpreting these naïve analyses owing to the absence of adjustment for bias. ≥*CR* complete response or better, CI, confidence interval, *Kd* carfilzomib and dexamethasone, *KM* Kaplan–Meier, *KRd* carfilzomib, lenalidomide and dexamethasone, *NE* non-estimable, *OS* overall survival, *PR* partial response, *VGPR* very good partial response.

Across all treatment arms in ASPIRE and ENDEAVOR, patients with a best response (≥CR subgroup) had longer median PFS and OS than those in the VGPR/PR subgroup (Figs. 1 and 2; Supplementary Figs. S1 and S2). However, irrespective of the response achieved, treatment with carfilzomib-based regimens (KRd and Kd) resulted in better PFS and OS than treatment with Rd or Vd, respectively (Supplementary Figs. S1 and S2). In the KRd arm of ASPIRE, for the ≥CR and VGPR/PR subgroups, respectively, median PFS was 50.4 (95% CI: 37.9, 64.6) months versus 22.1 (18.4, 24.1) months (Fig. 1a), and median OS was 67.0 (56.7, NE) versus 44.2 (36.2, 51.2) months (Fig. 2a). In the Kd arm of ENDEAVOR, for the ≥CR and VGPR/PR subgroups, respectively, median PFS was 34.0 (26.0, NE) versus 20.4 (17.0, 23.0) months (Fig. 1b), and median OS was non-estimable (NE) in each subgroup (Fig. 2b). Although fewer patients in the comparator arms (Rd and Vd) achieved ≥CR than those in the carfilzomib-based treatment arms (KRd and Kd), patients who achieved ≥CR in the comparator arms of both studies also experienced longer median PFS and OS compared with patients who achieved VGPR/PR (Supplementary Figs. S1 and S2).

### Exposure to study treatment analysis

Patient numbers in the safety populations of the carfilzomib arms of ASPIRE (KRd) and ENDEAVOR (Kd) were *n* = 392 and *n* = 463, respectively [11, 12]. In the KRd arm of ASPIRE, the median treatment duration (range) was 148.3 weeks (9.1–323.9) in the ≥CR subgroup (37 cycles; per protocol, carfilzomib was stopped after 18 cycles in ASPIRE) versus 78.7 weeks (7.1–33.9) in the VGPR/PR subgroup (19 cycles) (Table 2). In the Kd arm of ENDEAVOR, the median treatment duration was 72.0 weeks (16.0–238.9) in the ≥CR subgroup (17 cycles) versus 57.3 weeks (2.0–230.1) in the VGPR/PR subgroup (14 cycles) (Table 2).

**Table 2:** Exposure to study treatment among patients who achieved a best response (≥CR) versus VGPR/PR from the KRd arm of ASPIRE and the Kd arm of ENDEAVOR (safety populations).

ASPIRE, KRd^a^	≥CR	VGPR/PR
	*n* = 126	*n* = 219
Number of cycles started		
Mean (SD)	42.0 (21.97)	25.5 (21.02)
Median (range)	37.0 (3.0–80.0)	19.0 (2.0–82.0)
Treatment duration, weeks		
Mean (SD)	170.7 (88.9)	103.5 (85.23)
Median (range)	148.3 (9.1–323.9)	78.7 (7.1–333.9)
Cumulative dose of carfilzomib across cycles,^a^ mg/m^2^		
Mean (SD)	2356.5 (357.46)	1945.0 (719.76)
Median (range)	2470.0 (364.0–2902.0)	2344.0 (40.0–2578.0)

ENDEAVOR, Kd	*n* = 58	*n* = 298
Number of cycles patient dosed		
Mean (SD)	23.1 (15.7)	18.2 (12.9)
Median (range)	17.5 (4.0–59.0)	14.0 (1.0–58.0)
Treatment duration, weeks		
Mean (SD)	95.5 (65.2)	74.1 (53.4)
Median (range)	72.0 (16.0–238.9)	57.3 (2.0–230.1)
Cumulative dose of carfilzomib across cycles, mg/m^2^		
Mean (SD)	6745.3 (4703.5)	5221.2 (3808.5)
Median (range)	5014.8 (1262.5–17 713.7)	4185.4 (137.7–18 239.5)

*≥CR* complete response or better, *Kd* carfilzomib and dexamethasone, *KRd* carfilzomib, lenalidomide and dexamethasone, *PR* partial response, *SD* standard deviation, *VGPR* very good partial response.

^a^Per protocol, carfilzomib was withheld after 18 cycles in the ASPIRE study.

### Safety analysis

Despite a longer treatment duration of KRd patients in the ≥CR subgroup than in the VGPR/PR subgroup (148.3 vs 78.7 weeks, Table 2) in ASPIRE, both subgroups received a similar cumulative dose of carfilzomib that was stopped after 18 cycles. In this study, incidences of all TEAEs, and of grade 3 or higher TEAEs were comparable between the ≥CR and VGPR/PR subgroups, whereas incidences of serious TEAEs were higher in the ≥CR subgroup (Table 3). In ENDEAVOR, longer exposure to Kd in the ≥CR subgroup than in the VGPR/PR subgroup (median duration of treatment: 72.0 vs 57.3 weeks; median cumulative dose of carfilzomib: 5014.8 vs 4185.4 mg/m^2^; Table 2) was observed and was not associated with higher incidences of TEAEs. Exposure-unadjusted incidences of all TEAEs, and of grade 3 or higher TEAEs, including serious TEAEs, were similar between the ≥CR and VGPR/PR subgroups in ENDEAVOR (Table 3). The ratios of exposure-adjusted incidence rates indicated that there was no difference in the risk of TEAEs of interest between the ≥CR and VGPR/PR subgroups in each study (Table 4). Of note, fewer patients in the ≥CR subgroups than in the VGPR/PR subgroups experienced TEAEs that led to discontinuation of carfilzomib in ASPIRE or of Kd in ENDEAVOR (Table 3).

**Table 3:** Exposure-unadjusted incidence of TEAEs among patients who achieved a best response (≥CR) versus VGPR/PR from **a** and **b**.

(a) The KRd arm of ASPIRE (safety population)		
TEAE, *n* (%)	≥CR	VGPR/PR
ASPIRE, KRd	*n* = 126	*n* = 219
All	125 (99.2)	217 (99.1)
Grade ≥ 3	112 (88.9)	190 (86.8)
SAEs	92 (73.0)	134 (61.2)
Leading to discontinuation of carfilzomib	7 (5.6)	29 (13.2)
Leading to discontinuation of any study drug	54 (42.9)	62 (28.3)
Leading to discontinuation of all study drugs	27 (21.4)	36 (16.4)
Leading to death	15 (11.9)	20 (9.1)

(b) The Kd arm of ENDEAVOR (safety population)		
TEAE, *n* (%)	≥CR	VGPR/PR
ENDEAVOR, Kd	*n* = 58	*n* = 298
All	57 (98.3)	295 (99.0)
Grade ≥ 3	46 (79.3)	243 (81.5)
SAEs	30 (51.7)	177 (59.4)
Leading to study treatment discontinuation	15 (25.9)	90 (30.2)
Leading to death	1 (1.7)	13 (4.4)

*≥CR* complete response or better, *Kd* carfilzomib and dexamethasone, *KM* Kaplan–Meier, *KRd* carfilzomib, lenalidomide and dexamethasone, *PR* partial response, *SAE* serious adverse event, *TEAE* treatment-emergent adverse event, *VGPR* very good partial response.

**Table 4:** Exposure-adjusted incidence of grade 3 or higher TEAEs among patients who achieved a best response (≥CR) compared with VGPR/PR from **a** and **b**.

(a) The KRd arm of ASPIRE (safety population)							
ASPIRE, KRd	≥CR (*n* = 126)			VGPR/PR (*n* = 219)			CR + vs VGPR/PR
TEAE of interest of grade 3 or higher^a^	Total person time (years)^b^	Total number of patients with events, *n* (%)	Exposure-adjusted risk estimate per 100 patient years (95% CI)	Total person time (years)^b^	Total number of patients with events, *n* (%)	Exposure-adjusted risk estimate per 100 patient years (95% CI)	Risk Ratio (95% CI)CR + /(VGPR/PR)
Cardiac adverse events							
Cardiac failure (SMQN)	401.8	6 (4.8)	1.49 (0.67–3.32)	424.6	9 (4.1)	2.12 (1.10–4.07)	0.70 (0.25, 1.98)
Ischemic heart disease (SMQB)	405.4	3 (2.4)	0.74 (0.24–2.29)	427.7	8 (3.7)	1.87 (0.94–3.74)	0.40 (0.11, 1.49)
Hematologic adverse events							
Neutropenia (PT)	300.1	43 (34.1)	14.33 (10.63–19.32)	335.8	69 (31.5)	20.55 (16.23–26.01)	0.70 (0.48, 1.02)
Thrombocytopenia (PT)	351.2	23 (18.3)	6.55 (4.35–9.85)	391.1	35 (16.0)	8.95 (6.43–12.46)	0.73 (0.43, 1.23)
Anemia (PT)	366.7	22 (17.5)	6.00 (3.95–9.11)	394.7	39 (17.8)	9.88 (7.22–13.52)	0.61 (0.36, 1.02)
Neuropathy adverse events							
Peripheral neuropathy (SMQB)	398.9	7 (5.6)	1.75 (0.84, 3.68)	418.1	10 (4.6)	2.39 (1.29, 4.45)	0.73 (0.28, 1.93)
Pulmonary adverse events							
Dyspnea (PT)	404.2	4 (3.2)	0.99 (0.37–2.64)	426.8	5 (2.3)	1.17 (0.49–2.81)	0.84 (0.23, 3.15)
Renal adverse events							
Acute renal failure (SMQN)	402.9	6 (4.8)	1.49 (0.67–3.31)	423.8	8 (3.7)	1.89 (0.94–3.77)	0.79 (0.27, 2.27)
Thromboembolic adverse events							
Embolic and thrombotic events, venous (SMQN)	386.7	11 (8.7)	2.84 (1.58–5.14)	419.7	8 (3.7)	1.91 (0.95–3.81)	1.49 (0.60, 3.71)
Hypertension (PT)	389.2	9 (7.1)	2.31 (1.20–4.44)	415.1	11 (5.0)	2.65 (1.47–4.79)	0.87 (0.36, 2.11)

(b) The Kd arm of ENDEAVOR (safety population)							
ENDEAVOR, Kd	≥CR (*n* = 58)			VGPR/PR (*n* = 298)			CR + vs VGPR/PR
TEAE of interest^a^	Total person time (years)^b^	Total number of patients with events, *n* (%)	Exposure-adjusted risk estimate per 100 patient years (95% CI)	Total person time (years)^b^	Total number of patients with events, *n* (%)	Exposure-adjusted risk estimate per 100 patient years (95% CI)	Risk Ratio (95% CI) CR + /(VGPR/PR)
Cardiac adverse events							
Cardiac failure (SMQN)	105.3	1 (1.7)	0.95 (0.13–6.74)	413.6	20 (6.7)	4.84 (3.12–7.49)	0.20 (0.03, 1.46)
Ischemic heart disease (SMQB)	105.2	0 (0.0)	NE (NE–NE)	416.2	8 (2.7)	1.92 (0.96–3.84)	NE (NE, NE)
Hematologic adverse events							
Neutropenia (PT)	100.5	2 (3.4)	1.99 (0.50–7.96)	415.3	5 (1.7)	1.20 (0.50–2.89)	1.98 (0.86, 4.59)
Thrombocytopenia (PT)	97.5	8 (13.8)	8.21 (4.10–16.41)	410.7	17 (5.7)	4.14 (2.57–6.66)	0.47 (0.18, 1.18)
Anemia (PT)	99.1	5 (8.6)	5.05 (2.10–12.12)	387.8	42 (14.1)	10.83 (8.00–14.66)	1.65 (0.32, 8.52)
Neuropathy adverse events							
Peripheral neuropathy (SMQB)	102.1	4 (6.9)	3.92 (1.47–10.44)	403.0	15 (5.0)	3.72 (2.24–6.17)	1.05 (0.35, 3.17)
Pulmonary adverse events							
Dyspnea (PT)	99.9	4 (6.9)	4.01 (1.50–10.67)	401.6	18 (6.0)	4.48 (2.82–7.11)	0.89 (0.30, 2.64)
Renal adverse events							
Acute renal failure (SMQN)	105.2	0 (0.0)	NE (NE–NE)	412.4	22 (7.4)	5.34 (3.51–8.10)	NE (NE, NE)
Thromboembolic adverse events							
Embolic and thrombotic events, venous (SMQN)	100.3	2 (3.4)	1.99 (0.50–7.97)	405.0	12 (4.0)	2.96 (1.68–5.22)	0.67 (0.15, 3.00)
Hypertension (PT)	87.2	14 (24.1)	16.05 (9.51–27.10)	369.8	48 (16.1)	12.98 (9.78–17.23)	1.24 (0.68, 2.24)

*CI* confidence interval, ≥*CR* complete response or better, *HLT* high level term, *HLGT* high level group term, *Kd* carfilzomib and dexamethasone, *KRd* carfilzomib, lenalidomide and dexamethasone arm, *MedDRA* Medical Dictionary for Regulatory Activities, *NE* non-evaluable, *PR* partial response, *PT* preferred term, *SMQB* standardized MedDRA query, broad scope, *SMQN* standardized MedDRA query, narrow scope, *TEAE* treatment-emergent adverse event, *VGPR* very good partial response.

^a^TEAEs were defined as any adverse event with an onset date between the first dose and 30 days after the last dose of any study drug. TEAEs were coded using MedDRA version 20.0. Patients were counted only once for each event of interest.

^b^Total person time was the sum of time to first TEAE for all patients in each subgroup. For patients who did not experience a TEAE, the entire time exposed to study treatment was considered in the sum.

### Multivariate analysis for a best response (≥CR)

Among patients in ASPIRE and ENDEAVOR receiving carfilzomib, the multivariate analyses revealed evidence of an association (*p* < 0.05) with achieving ≥CR vs not achieving ≥CR for only a few factors, namely low LDH for patients in ASPIRE and a better ECOG performance for patients in ENDEAVOR (Table 5). Of note, no association was found between cytogenetic risk status at baseline (high vs standard risk/NR) and ≥CR (Table 5).

**Table 5:** Multivariate logistic regression analysis of the likelihood of a best response (≥CR) versus not a CR^a^ in patients from **a** and **b**.

(a) The KRd arm of ASPIRE			
Factor^b^	ASPIRE (KRd)		
	OR	95% CI	*p* value^c^
Age, years: ≥65 vs < 65	1.325	0.829–2.118	0.2402
ECOG PS, 1–2 vs 0	0.868	0.546–1.381	0.5510
LDH, U/l: >360 vs ≤ 360	0.516	0.289–0.920	0.0251
Cytogenetics: high vs standard risk/NR	1.033	0.502–2.128	0.9296
Serum calcium, mmol/l: >2.75 vs ≤ 2.75	1.197	0.273–5.247	0.8116
β2-microglobulin, mg/l: ≥3.5 vs < 3.5	0.879	0.534–1.445	0.6105
Serum albumin, g/dl: <3.5 vs ≥ 3.5	0.609	0.329–1.127	0.1145
Bone marrow plasma cell count, %: ≥70 vs < 70	1.103	0.500–2.432	0.8075
Thrombocyte count, 10^9^ cells/l: ≤100 vs > 100	0.701	0.302–1.630	0.4093
Refractory to immediately previous regimen: yes vs no	0.735	0.436–1.238	0.2473
Number of previous lines of treatment: ≥2 vs 1	0.930	0.586–1.477	0.7590

(b) The Kd arm of ENDEAVOR			
Factor^d^	ENDEAVOR (Kd)		
	OR	95% CI	*p* value^c^
Age, years: ≥65 vs < 65	0.604	0.328–1.109	0.1040
ECOG PS, 1–2 vs 0	0.462	0.248–0.860	0.0148
Cytogenetics: high vs standard risk/NR	1.306	0.663–2.573	0.4405
Serum calcium, mmol/l: >2.75 vs ≤ 2.75	1.896	0.372–9.673	0.4416
β2-microglobulin, mg/l: ≥3.5 vs < 3.5	0.796	0.414–1.529	0.4934
Serum albumin, g/dl: <3.5 vs ≥ 3.5	0.788	0.279–2.220	0.6517
Bone marrow plasma cell count, %: ≥70 vs < 70	1.273	0.442–3.668	0.6545
Thrombocyte count, 10^**9**^ cells/l: ≤100 vs > 100	1.179	0.417–3.338	0.7560
Refractory to immediately previous regimen: yes vs no	0.559	0.286–1.096	0.0903
Number of previous lines of treatment: ≥2 vs 1	1.484	0.803–2.744	0.2078

*CI* confidence interval, ≥*CR* complete response or better, *ECOG* Eastern Cooperative Oncology Group, *Kd* carfilzomib and dexamethasone, *KRd* carfilzomib, lenalidomide and dexamethasone, *LDH* serum lactate dehydrogenase, *NR* not reported, *OR* odds ratio, *PR* partial response, *VGPR* very good partial response.

^a^A non-best response (not a CR) was defined as VGPR, PR, minimal response (MR), stable disease or progressive disease (PD).

^b^The factors were treated as categorical variables in the models and no imputation for missing data was performed. The models included the factors as independent variables and ≥CR as the dependent variable, and a threshold of *p* < 0.05 was specified for suggesting evidence of an association between each variable and achieving a ≥CR.

^c^A threshold of *p* < 0.05 was specified for suggesting evidence of an association between each variable and ≥CR.

^d^No patients in the K^d^ arm of ENDEAVOR had serum LDH > 360 U/l.

## Discussion

These post hoc exploratory analyses of extended follow-up data from the ASPIRE and ENDEAVOR pivotal phase 3 studies of carfilzomib in RRMM showed that best responses (≥CR) were achieved by 31.8 and 13.0% of patients treated with KRd and Kd, respectively. Most patients in the ≥CR subgroups achieved this response early: the median time to ≥CR was 6.7 months (min, max: 1.4, 30.2 months) for patients treated with KRd in ASPIRE [15] and 6.8 months (min, max: 2.0, 20.8 months) for those treated with Kd in ENDEAVOR. Best responses translated into better survival outcomes than those observed in patients who achieved VGPR/PR, and our multivariate regression results suggested that best responses to carfilzomib-based treatment may be achieved irrespective of most patient baseline and disease characteristics evaluated in the models, including cytogenetic risk status.

Because patients who achieved ≥CR in both arms of ASPIRE and ENDEAVOR benefited from improvements in PFS and OS compared with those who achieved VGPR/PR, our data suggest that an important consideration for improving survival outcomes should be the achievement of a deep response to a given treatment regimen. Consistent with our results, published studies have generally confirmed an association between better responses and improved survival outcomes in patients with either newly diagnosed MM or relapsed MM. In a post hoc analysis from ASPIRE, carfilzomib provided greater responses and improvements in PFS at 18 months and cumulative ≥CR rates increased over time suggesting that there may benefit of continued carfilzomib treatment [15]. However, the degree of association may be different depending on patient and disease factors, and specific treatments received [34–37]. A large meta-analysis of data from 102 studies involving 13 322 patients with RRMM indicated a correlation between the rate of VGPR or better and median PFS (*R*^2^ = 0.63) [38].

In the present study, best responders to carfilzomib-based treatment had longer treatment durations on average than individuals who achieved VGPR/PR in both ASPIRE and ENDEAVOR. Treatment with carfilzomib in ASPIRE was stopped after 18 cycles, while those treated with Kd in ENDEAVOR were treated until progression. The maximum time to ≥CR for patients treated with KRd in ASPIRE was 30.2 months and for those treated with Kd in ENDEAVOR was 20.8 months suggesting that treatment continuation until progression can lead to deeper responses over time. Indeed, published data have associated longer MM treatment durations with improved survival outcomes [39–41]. The relationship between treatment duration and response may be confounded, however, by the fact that patients who are able to tolerate the regimen are likely to stay on treatment and improve the response over time. Furthermore, despite being treated for longer than those who achieved VGPR/PR, patients who achieved ≥CR were less likely to experience TEAEs that led to discontinuation of treatment with carfilzomib in ASPIRE or with Kd in ENDEAVOR. These observations could also reflect the suggestion that best responders may be fitter at baseline, and therefore may be able to tolerate longer treatment durations than patients who achieved VGPR/PR [15]. In an updated analysis of ENDEAVOR, it was observed that the incidence of grade 3 or higher TEAEs decreased overtime in both Kd and Vd arms. Overall, 74.1% of patients experienced grade 3 or higher TEAEs in the first 12 months and this decreased to 54% when measured at 36 months [13]. Therefore, in real-world practice, appropriate management of TEAEs may help to prevent discontinuation of carfilzomib treatment and provide an opportunity to improve depth of response over time.

Baseline characteristics were generally similar for patients who achieved a best response and those who achieved VGPR/PR with carfilzomib-based treatment, although there were some differences such as ECOG performance status zero. In ENDEAVOR, patients who achieved ≥CR were slightly younger on average, and more had undergone HSCT previously than those who achieved VGPR/PR. These patient characteristics also corroborate prior observations that best responders to carfilzomib-based treatment in ASPIRE and ENDEAVOR were somewhat fitter at baseline than those who achieved VGPR/PR. Of note, frailty has been associated with inferior outcomes and poorer survival in patients with multiple myeloma [42].

In our multivariate regression analysis, low serum LDH was the only factor associated with the incidence of ≥CR in ASPIRE. Interestingly, no association was found between cytogenetic risk status and ≥CR. Similarly, in a preplanned subgroup analysis of ENDEAVOR evaluating Kd vs Vd by cytogenetic risk, no association was observed between cytogenetic risk status and ≥CR. In the high-risk group, 15.5% of patients treated with carfilzomib achieved ≥CR in comparison to 13.0% of patients achieving ≥CR in the standard-risk group [20]. However, in a preplanned subgroup analyses of ASPIRE, 29.2% of patients with high-risk cytogenetics receiving a carfilzomib-based treatment achieved ≥CR in comparison to 38.1% of patients with standard risk [43]. These data suggest that high-risk cytogenetics may not have an important influence on achieving best responses of ≥CR to carfilzomib-based treatment. It would be of interest to further explore with more sensitive techniques such as minimal residual disease (MRD) the association between cytogenetics and survival outcomes that were found to be poorer for ≥CR patients with high-risk cytogenetics compared with those with standard risk in both ENDEAVOR and ASPIRE [13].

### Limitations

No adjustment for bias was applied to the KM analyses of PFS and OS by response status. Bias may have resulted from the requirement for best responders to survive long enough to achieve ≥CR, therefore caution is warranted when interpreting the results from these naïve KM analyses. As with all post hoc modeling, our analyses are exploratory and no provision was made for adequate sample sizes to detect significant associations in the data. Furthermore, there is a risk of spurious associations, which was minimized to some extent by using a recently published risk stratification algorithm [44] that has been validated in real world populations [32, 44] to inform the selection of variables and threshold values for the multivariate regression models.

An additional limitation of the study was the absence of a MRD subgroup in the regression analyses. It would be of interest in future studies to perform a similar predictive analysis including a MRD-negative subgroup of patients. Given the high CR rates now achievable with modern MM therapies, including carfilzomib-based regimens [4, 7, 10], assessments of deeper responses to treatment, such as stringent CR (sCR) or conversion to MRD-negativity, will become increasingly important [31, 45, 46]. sCR was achieved in 56 patients in the KRd arm of ASPIRE and 8 patients in the Kd arm of ENDEAVOR at the respective interim analyses with some possibly achieving MRD-negativity [11, 12].

## Conclusions

Treatment with KRd and Kd may lead to rapid and deep responses irrespective of most patient baseline and disease characteristics. Best responders presented a longer exposure to carfilzomib and had longer median PFS and OS than patients who achieved VGPR/PR in both studies.

## Supplementary information

Supplementary file information

Supplementary Fig 1

Supplementary Fig 2
