# Supplementary material for: Efficacy and safety profile of deep responders to carfilzomib-based therapy: a subgroup analysis from ASPIRE and ENDEAVOR
Source: Leukemia. 2020 Oct 16;35(6):1732–44. doi: 10.1038/s41375-020-01049-5 (PMC8179852; doi:10.1038/s41375-020-01049-5)
Supplement: Supplementary file 1 — Supplementary file information [file 41375_2020_1049_MOESM1_ESM.docx]

**Supplementary information**

**Supplementary file 1**

Supplementary Fig. 1: Kaplan-Meier curves for progression-free survival for patients in the comparator arms of ASPIRE (Rd) and ENDEAVOR (Vd)

**Supplementary file 2**

Supplementary Fig. 2: Kaplan-Meier curves for overall survival for patients in the comparator arms of ASPIRE (Rd) and ENDEAVOR (Vd)

**Supplementary Fig. 1** KM curves for PFS for patients who achieved a best response (≥CR) versus those who achieved VGPR/PR from **a** the Rd arm of ASPIRE and **b** the Vd arm of ENDEAVOR

*≥CR* complete response or better, *KM* Kaplan–Meier, *PFS* progression-free survival, *PR* partial response, *Rd* lenalidomide and dexamethasone, *Vd* bortezomib and dexamethasone, *VGPR* very good partial response

**Supplementary Fig. 2** KM curves for OS for patients who achieved a best response (≥CR) versus those who achieved VGPR/PR from **a** the Rd arm of ASPIRE and **b** the Vd arm of ENDEAVOR.

*CR* complete response or better, *KM* Kaplan–Meier, *NE* not evaluable, *OS* overall survival, *PR* partial response, *Rd* lenalidomide and dexamethasone, *Vd* bortezomib and dexamethasone, *VGPR* very good partial response
